# Beyond avatars and arrows: Testing the mentalising and submentalising hypotheses with a novel entity paradigm

**DOI:** 10.1177/17470218211007388

**Published:** 2021-04-13

**Authors:** Evan Westra, Brandon F Terrizzi, Simon T van Baal, Jonathan S Beier, John Michael

**Affiliations:** 1 Department of Philosophy, York University, Toronto, Ontario, Canada; 2 Division of General and Community Pediatrics, Cincinnati Children’s Hospital Medical Center, Cincinnati, OH, USA; 3 Department of Philosophy, Monash University, Clayton, Victoria, Australia; 4 Department of Cognitive Science, Central European University, Budapest, Hungary

**Keywords:** Perspective-taking, animacy attribution, submentalising, dot-perspective task

## Abstract

In recent years, there has been a heated debate about how to interpret findings that seem to show that humans rapidly and automatically calculate the visual perspectives of others. In this study, we investigated the question of whether automatic interference effects found in the dot-perspective task are the product of domain-specific perspective-taking processes or of domain-general “submentalising” processes. Previous attempts to address this question have done so by implementing inanimate controls, such as arrows, as stimuli. The rationale for this is that submentalising processes that respond to directionality should be engaged by such stimuli, whereas domain-specific perspective-taking mechanisms, if they exist, should not. These previous attempts have been limited, however, by the *implied intentionality* of the stimuli they have used (e.g., arrows), which may have invited participants to imbue them with perspectival agency. Drawing inspiration from “novel entity” paradigms from infant gaze–following research, we designed a version of the dot-perspective task that allowed us to precisely control whether a central stimulus was viewed as animate or inanimate. Across four experiments, we found no evidence that automatic “perspective-taking” effects in the dot-perspective task are modulated by beliefs about the animacy of the central stimulus. Our results also suggest that these effects may be due to the task-switching elements of the dot-perspective paradigm, rather than automatic directional orienting. Together, these results indicate that neither the perspective-taking nor the standard submentalising interpretations of the dot-perspective task are fully correct.

## Introduction

Mindreading—the ability to predict and interpret the behaviour of others in terms of their underlying mental states—is widely believed to be a central part of human social cognition (Apperly, 2011; Baron-Cohen, 1997; Spaulding, 2018; Tomasello, 2014; Wellman, 2014). However, cognitive scientists are divided about the psychological scope of mindreading and the range of cognitive phenomena that actually involve reasoning about unobservable mental states (Andrews, 2012; Bermudez, 2003; Heyes, 2014; Ruffman, 2014). Few would deny, of course, that the ability to represent mental states is required in certain complex tasks, such as making explicit verbal predictions about the actions of an agent with a false belief (Wellman et al., 2001; Wimmer & Perner, 1983). However, there is much more disagreement about whether more subtle instances of putative mentalising involve rapid, unconscious processing. This is because many of the tasks used to detect these subtle forms of mindreading also admit of lower level, non-mentalistic interpretations involving processes that only give the appearance of mental-state attribution—what Cecilia Heyes (2014, 2018) has called “submentalising.” If it were the case that many putatively mentalistic processes are in fact the product of submentalising, this would force many mindreading theorists to radically rethink widespread ideas about the scope of mindreading in everyday social cognition.

One paradigm that has become a particular focus of the mentalising-submentalising debate is the dot-perspective task, which was originally designed to determine whether adults spontaneously and unintentionally represent what others can or cannot see (Qureshi et al., 2010; Samson et al., 2010; Surtees et al., 2016). In standard versions of the dot-perspective task, participants are shown images of a room with three walls and a centrally placed human avatar standing in profile. In different trials, between zero and three red dots appear on the walls, while the avatar periodically changes its orientation. In some trials, participants must report the number of dots that they can see (i.e., “Self” trials), whereas in others, they must report the number of dots that the avatar can see (i.e., “Other” trials). The trials of interest are those where the number of dots that the avatar can see is inconsistent with the number of dots the participant can see, and the participant must report on their own perspective. On these trials, the avatar’s inconsistent perspective appears to interfere with the participant’s performance, leading to longer response times and increased rates of error.

Originally, this “altercentric interference” effect was interpreted as evidence that participants automatically represent the visual perspective of the avatars, even on trials where this information is irrelevant (Apperly & Butterfill, 2009; Qureshi et al., 2010; Samson et al., 2010). However, an alternative “submentalising” interpretation has also been offered: Participants are not in fact representing what the avatar can see, but are instead being spatially cued by the low-level directional properties of the avatar, which interfere with their performance on the task (Santiesteban et al., 2014). Thus, according to the submentalising interpretation, what drives the altercentric interference effect is not the visual perspective of the avatar but, rather, the fact that the avatar has a canonical orientation that draws our attention in a particular direction.

A number of experiments have been conducted to test these competing perspective-taking and submentalising hypotheses by holding fixed the directional characteristics of the avatar while manipulating the presence or absence of perspectival mental states. However, the results of these experiments have been somewhat equivocal. For example, Furlanetto and colleagues (2016) conducted a version of the dot-perspective task in which the avatar was shown wearing coloured goggles, which participants believed to be either transparent (the Seeing condition) or opaque (the Non-Seeing condition). Consistent with the perspective-taking hypothesis, they found evidence for altercentric inference in the Seeing condition trials, but not in Non-Seeing trials. However, Conway and colleagues (2017) failed to obtain a similar result in both exact and conceptual replications of this paradigm; instead, their results were broadly consistent with the submentalising hypothesis.

In what is perhaps the best-known test of the perspective-taking and submentalising hypotheses, Santiesteban and colleagues (2014) conducted another version of the dot-perspective task in which the effects of the avatar were compared with the effects of an arrow placed in the same location pointing towards one of the walls. They reasoned that if it were only the low-level directional properties of the avatar that drove the altercentric interference effect, then the presence of the arrow—a directional but otherwise inanimate stimulus—should yield a comparable result. This is indeed what they found, suggesting that it was the directionality of the avatar that drove the altercentric interference effect, rather than any of the intentional mental properties that people may have ascribed to it (see also O’Grady et al., 2017, and Santiesteban et al., 2017, for similar results).

However, in their original discussion of this experiment, Santiesteban and colleagues acknowledge that these results also lend themselves to an alternative, mentalistic interpretation—namely, that participants’ everyday experiences with arrows have led them to habitually attribute quasi-visual perspectives to them. This possibility is not particularly far-fetched: Dating back to Heider and Simmel’s famous intentionality attribution experiments, there is a large literature showing that humans both explicitly and implicitly attribute psychological properties to inanimate objects (Gao et al., 2009; Heider & Simmel, 1944). Arrows are particularly apt targets for this kind of attribution because they possess *derived intentionality* (Searle, 1983): They are imbued with semantic content by their authors and are used for specific communicative purposes—whether that is directing traffic, indicating salient locations on a map, or simply telling someone to “look over there!” In effect, they serve the same basic function as more naturalistic gestures, such as finger-pointing, nodding one’s head in a particular direction, or even visibly shifting one’s eyes. In other words, *both* arrows *and* avatars may confound intentionality and directionality.

Because of their mentalistic connotations, arrows are imperfect inanimate controls for the dot-perspective task. Santiesteban and colleagues tacitly acknowledge this confound, but they argue that such an interpretation would render the implicit mentalising hypothesis untestable in practice: If implicit perspective-taking were so promiscuous that it could occur even with inanimate entities, then it is not obvious how one could ever develop an adequate way to control for the role of perceived animacy in the dot-perspective task.

Thus, one of the fundamental challenges to resolving this debate about the dot-perspective task is to find a way to isolate the respective roles of abstract representations of perspectival agency and low-level directionality in a way that does not confound the two. This is the challenge we take on in this study. Our primary innovation is to replace both the avatars and the arrows employed in previous versions of the task with a single perceptually unfamiliar object and to then manipulate whether participants believe that the object is either animate or inanimate. This approach eliminates the possibility that participants in the Inanimate condition have prior mentalistic associations with the stimulus and also permits us to control for any other low-level perceptual differences between the Animate and Inanimate conditions.

Our approach is inspired by the use of “novel entity” paradigms in the study of infant gaze following (Beier & Carey, 2014; Johnson, 2003; Johnson et al., 1998, 2008). In these studies, 12-month-old infants will follow the implied attentional direction (i.e., “gaze”) of a novel object of ambiguous animacy provided that the object exhibits certain socially relevant behaviours, such as the capacity to respond to people in a contingent, communicative fashion. Once a novel object has been construed as a potential agent, infants and adults respond to it with a range of overt social behaviours, including following its gaze, describing its movements with intentionally laden vocabulary, and even helping it achieve an instrumental goal (Kenward & Gredebäck, 2013). However, neither gaze following nor intentional attributions occur if participants do not view the object’s initial behaviour as sufficiently social (Beier & Carey, 2014; Tauzin & Gergely, 2019).

Of particular relevance to the mentalising-submentalising debate surrounding the dot-perspective task, construing a novel object as an agent also influences more low-level, automatic processes. Terrizzi and Beier (2016) investigated the influence of agency construal on children’s and adults’ covert attentional responses within a gaze-cueing paradigm (Posner, 1980; Terrizzi & Beier, 2016). They found that participants fixated more quickly on laterally presented targets that appeared in locations congruent with the prior orientation of a novel object—but only when that object had previously behaved in a socially contingent manner indicative of its agency. In other words, the presence of social contingency information engages both overt and covert processes that underlie social interactions with others. These results are especially relevant because there is good reason to believe that both the gaze-cueing and dot-perspective tasks tap into similar underlying attentional processes (Bukowski et al., 2015; Westra, 2017). Thus, the employment of novel entities provides an opportunity to manipulate perceptions of animacy in a dot-perspective task.

In the four experiments reported here, we implemented a novel version of the dot-perspective task that replaced the central figure with an unfamiliar object modelled after the stimuli from Terrizzi and Beier (2016). To minimise the risk that participants would spontaneously imbue this central figure with perspectival agency, we ensured that it lacked any characteristically agentic features. Prior to completing the task, participants completed a familiarisation task in which participants either (a) read a story describing the unfamiliar object as though it were an animate agent or (b) were led to believe that the object was a completely inanimate entity with one side designated as its “front.” This enabled us to control whether the very same stimulus would be perceived as either an animate agent with an implied perspective or as an inanimate object with directional properties. Using unfamiliar objects also made it unlikely that participants would spontaneously imbue the central stimulus with perspectival agency, and thus helped to control for the confounds posed by the use of arrows as inanimate controls (cf. Santiesteban et al., 2014).

As with other versions of the dot-perspective task, we were most interested in “Self” trials in which the “perspective” of the stimulus was inconsistent with that of the participant. In Experiment 1, we aimed to determine whether participants exposed to our animacy familiarisation would subsequently display an altercentric interference effect on these trials comparable to the one that is usually observed in other versions of the dot-perspective task (Qureshi et al., 2010; Samson et al., 2010; Santiesteban et al., 2014).

In Experiment 2, we employed a between-subjects animacy manipulation to test whether any altercentric interference effect may be due to participants’ perception of the unfamiliar object as an agent or as an inanimate entity with a front. If the mentalistic interpretation of the dot-perspective task is correct, we predicted that only participants instructed to view the entity as an agent (an alien creature) should display the altercentric interference effect, because this is the only condition in which the central stimulus is presented as having a perspective. If, however, the submentalising interpretation of the task is correct, participants instructed to view the entity as an inanimate object (a mineral) should also display such an effect.

Experiment 3 was designed to overcome one limitation of Experiment 2. Specifically, in Experiment 2, participants saw both the animate alien and inanimate mineral, which were highly visually similar to one another. This might have caused participants in the Inanimate condition to mistake the mineral for the alien, causing them to view the former as animate. To probe this possibility, Experiment 3 implemented a different familiarisation phase which did not introduce the possibility that the entity may be an animate agent.

Experiment 4 employed the same between-subjects animacy manipulation as Experiment 2 with one important difference: Participants were only ever asked to report the number of dots visible from their own perspective, as opposed to the “perspective” of the novel entity. This design enabled us to perform a further test of the perspective-taking hypothesis while also allowing us to tease apart the predictions of the submentalising account from an alternative non-mentalistic interpretation based on the task-switching demands of the dot-perspective paradigm.

## Experiment 1

The hypotheses, sample sizes, methods, and initial analyses were all pre-registered before data collection. The pre-registration and the script used for this experiment, as well as all the raw data, can be accessed at https://osf.io/pm3gu.

### Participants

Using G*Power 3.1 (Faul et al., 2009), we determined that a sample size of 34 would provide 80% statistical power for detecting a medium-sized effect (*d* = 0.5) in the planned comparisons, assuming a paired (two-tailed) *t*-test with an alpha level of .05. Due to experimenter error, we collected 37 rather than 34 participants. Four participants were excluded because their overall accuracy was below the threshold of 85%. Thus, the final sample was made up of 33 participants (15 males, 18 females, 0 undisclosed, *M_age_* = 26.15 years, *SD_age_* = 4.96 years). For recruitment, we used the participant database at the University of Warwick, where the experiment was conducted. All participants were naïve to the purpose of the study, reported normal or corrected-to-normal vision, and signed informed consent prior to the experiment. The experiment was conducted in accordance with the Declaration of Helsinki and was approved by the Humanities and Social Sciences Research Ethics Committee at the University of Warwick. Each participant received £6 for participating.

### Apparatus and stimuli

The experiment was displayed on a 13-inch computer screen (resolution: 2,560 × 1,600 pixels, refresh rate: 60 Hz). The programme for the experiment was written in Python (Peirce, 2007), with a frame rate of 17 frames per second.

As in Samson et al. (2010), the stimuli consisted of a picture showing a lateral view into a room with the left, back, and right walls visible; on test trials, zero, one, or two red dots were displayed in either one or two walls. During the familiarisation phase, participants saw images of a novel entity (based on the novel entity in Terrizzi & Beier, 2016) positioned in the centre of the room, with text representing the speech of the entity appearing above the room against the grey background.

### Procedure

After giving their informed written consent and reading the instructions, participants underwent a familiarisation phase. During this phase, they clicked through a succession of slides in which the novel entity introduced itself as a member of an alien species named “Dax” and described the life habits of this species. Participants then performed a brief task in which they differentiated between the novel entity and a differently coloured version of the novel entity, which was described as a “mineral” (this aspect of the familiarisation was included in anticipation of the animacy manipulation in Experiment 2). Familiarisations can be viewed at https://osf.io/9ghvs/.

**Figure 1.**
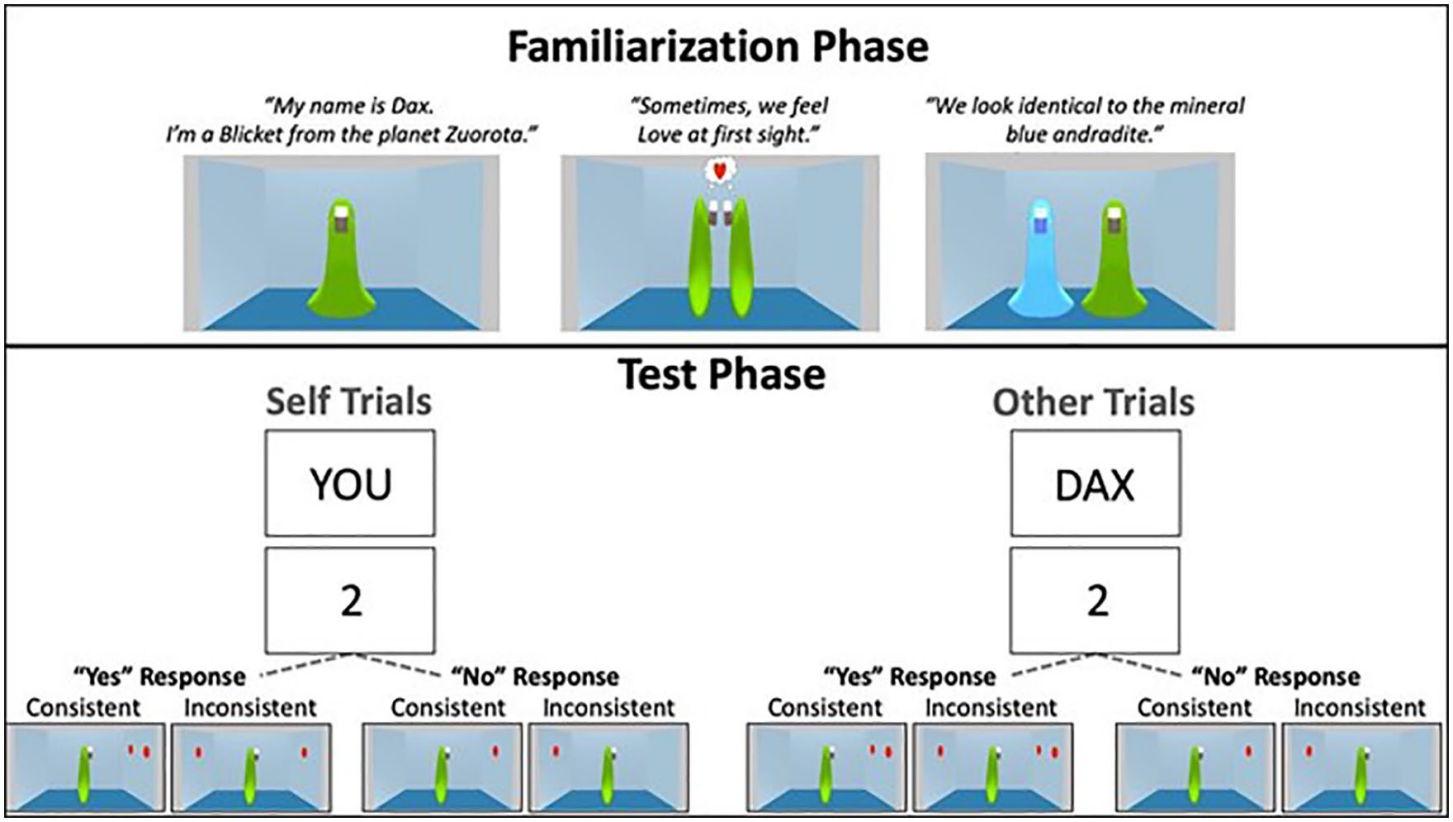
Schematic depiction of familiarisation and test phase procedures from Experiment 1. On matching trials (“yes” response) in the test phase, the digit specifying the target number corresponded to the number of dots on the walls. On mismatching trials (“no” response) in the test phase, the digit specified a number that was either one higher or one lower than the number of dots on the walls. On Inconsistent trials, the number of dots on the wall matched for one perspective, but not for the other. On Consistent trials, the number of dots matched both perspectives.

During the test trials (see Figure 1), a fixation cross was displayed for 500 ms, followed by a 750-ms presentation of either the word “YOU” or the word “DAX,” which specified whether the participant had to judge their own perspective or the perspective of the novel entity (i.e., a Self trial or an Other trial). Next, a digit (0–3) appeared for 750 ms, which specified a target number of dots for the participant to verify. The image of the room then appeared with the dots on the walls and the novel entity avatar in the centre. The dots remained on the screen until a response was given or 2,000 ms elapsed, whereupon the next trial would begin.

Participants then completed four blocks, each consisting of 80 trials. Of these 80 trials, 8 were fillers (i.e., the correct answer is “0”). Of the 72 test trials, the correct answer to 36 of them was “yes” and the correct answer to 36 of them was “no.” Of the 36 test trials analysed from each block, 18 were Self-perspective trials and 18 were Other-perspective trials. As in previous studies using this paradigm, we will only analyse trials for which the correct answer is “yes” (Michael et al., 2018; Samson et al., 2010). This is because the non-matching trials have to be constructed in an unbalanced manner: On mismatching (“no” response) consistent trials, the digit specifies a number of discs that did not correspond to anyone’s perspective, making these trials particularly easy to process.

### Results and discussion

To control for speed-accuracy trade-offs, reaction time (RT) for correct responses and hit rates (HRs) were merged into inverse efficiency scores (IESs), a combined measure which homogenises different patterns of speed-accuracy trade-offs within a group (IES = RT/HR; Townsend & Ashby, 1978). As the calculation of IES entails that RTs are quasi-exponentially multiplied as the HR decreases, Bruyer and Brysbaert (2011) have recommend not using the IES unless the mean HR within a group is above 90%, and HRs are negatively correlated with RTs. In our sample, the mean HR was above 90% in each experiment, and HRs were negatively correlated with RTs. A negative correlation between HR and RT implies a lack of speed-accuracy trade-offs, which indicates that it was appropriate to use IES for the primary analysis. This negative correlation between HR and RT was present in each experiment (Experiment 1: *r* = −.20, Experiment 2: *r* = −.18, Experiment 3: *r* = −.34, Experiment 4: *r* *=* −.31). We also include a table of the RTs and HRs in each condition for all three experiments (Table 1). In calculating mean RTs, response omissions due to the timeout procedure (1.16% of the data) and erroneous responses (3.06% of the data) were eliminated, as were trials where the correct response was “no” (50%). We also removed trials with responses that were more than 2.5 *SD*s greater or less than the mean for each participant for each condition (2.57% of the data).

**Table 1.** Descriptive statistics.

(A) IESs							
Consistency	Perspective	Exp. 1	Exp. 2		Exp. 3	Exp. 4	
		(Inanimate)	Animate	Inanimate	(Inanimate)	Animate	Inanimate
Consistent	Self	765 (170)	775 (156)	784 (207)	733 (123)	699 (116)	832 (149)
	Other	754 (157)	799 (179)	796 (189)	749 (130)		
Inconsistent	Self	785 (164)	831 (177)	841 (190)	777 (114)	709 (118)	832 (137)
	Other	839 (170)	914 (195)	929 (187)	861 (159)		
(B) RTs							
Consistency	Perspective	Exp. 1	Exp. 2		Exp. 3	Exp. 4	
		(Inanimate)	Animate	Inanimate	(Inanimate)	Animate	Inanimate
Consistent	Self	747 (140)	764 (150)	758 (154)	717 (110)	686 (112)	795 (116)
	Other	749 (152)	781 (156)	778 (169)	732 (110)		
Inconsistent	Self	757 (148)	790 (154)	786 (165)	736 (100)	687 (112)	798 (106)
	Other	798 (153)	833 (161)	847 (188)	795 (119)		
(C) HRs							
Consistency	Perspective	Exp. 1	Exp. 2		Exp. 3	Exp. 4	
		(Inanimate)	Animate	Inanimate	(Inanimate)	Animate	Inanimate
Consistent	Self	0.98 (0.03)	0.99 (0.03)	0.98 (0.06)	0.98 (0.03)	.98 (.03)	.96 (.03)
	Other	0.99 (0.02)	0.98 (0.03)	0.98 (0.03)	0.98 (0.04)		
Inconsistent	Self	0.97 (0.04)	0.95 (0.04)	0.94 (0.06)	0.95 (0.04)	.96 (.04)	.96 (.04)
	Other	0.95 (0.05)	0.91 (0.07)	0.91 (0.06)	0.93 (0.06)		

Overview of (A) inverse efficiency scores (IESs), (B) reaction times (RTs), and (C) hit rates (HRs) and in each condition for all four experiments. The numbers in parentheses represent standard deviations.

**Figure 2.**
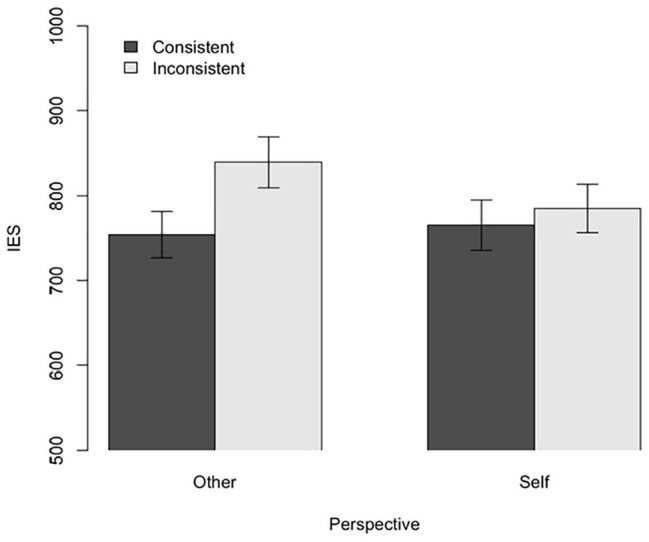
Experiment 1: Results for IES. Error bars indicate standard error.

We performed a two-way analysis of variance (ANOVA) for IES (see Figure 2). The results revealed a significant main effect of consistency, with performance being better in the Consistent condition (*M* = 759.75, *SD* = 162.93) than in the Inconsistent condition (*M* = 812.17, *SD* = 168.40), *F*(1, 32) = 48.01, *p* < .001, $\eta_{p}^{2}=.025$ . There was no main effect of perspective—that is, performance did not differ significantly between the self (*M* = 775.26, *SD* = 166.45) and the other (*M* = 796.65, *SD* = 168.41), *F*(2, 18) = 2.68, *p* = .111, $\eta_{p}^{2}=.004$ . We also observed an interaction between perspective and consistency, *F*(2, 18) = 28.36, *p* *<* .001, $\eta_{p}^{2}=.010$ .

Planned contrast analyses revealed that the difference in performance between consistent and inconsistent trials was significant when the task was to report what was in front of the novel entity, *t* (32) = 8.80, *p* < .001, *d* = 0.50; this provides evidence of egocentric interference. The difference in performance between consistent and inconsistent trials was marginally significant when the task was to report the content of their own perspective, *t*(32) = 2.01, *p* = .052, *d* = 0.18. These results indicate that the main effect of consistency was driven largely by egocentric interference, although the pattern of responses suggests a role for altercentric interference as well. We followed up on this pattern in Experiment 2.

## Experiment 2

In Experiment 1, we observed participant responses suggestive of an altercentric interference effect. Experiment 2 sought to replicate this finding and also investigate whether this pattern was driven primarily by the fact that the novel entity was presented as an animate agent or by its lower level directional properties. To this end, we employed a between-subjects design to compare responses to the novel entity across an Animate and Inanimate condition. Both conditions began with the same familiarisation sequence, also used in Experiment 1. This familiarisation introduced a novel alien creature and a novel mineral formation that was visually identical to the alien, save for a difference in colouring. In the Animate condition, the entity featured in the test trials was the alien/agent; thus, this condition was an internal replication of Experiment 1. In the Inanimate condition, the entity featured in the test trials was the mineral/inanimate object (which had been given an arbitrarily designated “front” during the familiarisation). Thus, the only major difference between the two conditions was whether participants believed that the novel entity before them during test trials was animate or inanimate. Both familiarisations can be viewed at https://osf.io/9ghvs/.

We predicted that if the effect of consistency we observed in Experiment 1 was driven by representations of animacy, we would observe it in the Animate condition only, or else that we would observe a larger effect of consistency in the Animate condition. If, however, the effects in Experiment 1 were in fact driven by the directionality of the novel entity, then we should instead find similarly sized effects of consistency in the Animate and Inanimate conditions. The hypotheses, sample sizes, methods, and initial analyses were all pre-registered before data collection. The pre-registration and the script used for this experiment, as well as all the raw data, can be accessed at https://osf.io/rg8st.

### Participants

To facilitate comparison with the results of Experiment 1, we aimed to include the same number of participants in each of the two groups in Experiment 2 as we had tested in Experiment 1. We therefore determined that the appropriate sample would be 74 participants (37 in each group). We collected data from 108 participants, and 30 were excluded because their overall accuracy was below the threshold of 85%.^1^ Thus, the final sample was made up of 78 participants (24 males, 54 females, 0 undisclosed, *M_age_* = 23.46 years, *SD_age_* = 5.58 years). For recruitment, we used the participant database at the Central European University, where the experiment was conducted. All participants were naïve to the purpose of the study, reported normal or corrected-to-normal vision, and signed informed consent prior to the experiment. The experiment was conducted in accordance with the Declaration of Helsinki and was approved by United Ethical Review Board for Research in Psychology (EPKEB). Each participant received gift vouchers totalling equivalent to €6 for participating.

### Apparatus and stimuli

The apparatus and stimuli were the same as in Experiment 1, with one exception: Test trials could feature either a blue or a green version of the novel entity.

### Procedure

The procedure was the same as in Experiment 1 except that one group of participants (Inanimate condition) were informed that the entity in the virtual room was a mineral, and a separate group was informed that it was an animate agent (Animate condition), as in Experiment 1. Within each group, half of the participants viewed a blue version of the novel entity, whereas the other half of the participants viewed a green version. Participants in the Animate condition were asked to respond based on the number of dots the novel entity could “see,” whereas participants in the Inanimate condition were asked to respond based on the number of dots that were “in front of” the novel entity. Before “Other” trials, participants in the Inanimate condition saw the word “MINERAL” instead of the word “DAX.”

### Results and discussion

As in Experiment 1, the mean HR in our sample was above 90% in all four conditions, indicating that it was appropriate to perform the analyses using IES. In calculating mean RTs, response omissions due to the timeout procedure (2.54% of the data) and erroneous responses (5.21% of the data) were eliminated from the data set, as were trials where the correct response was “no” (50%). We also removed trials with responses that were more than 2.5 *SD*s greater or less than the mean for each participant for each condition (2.44% of the data).

**Figure 3.**
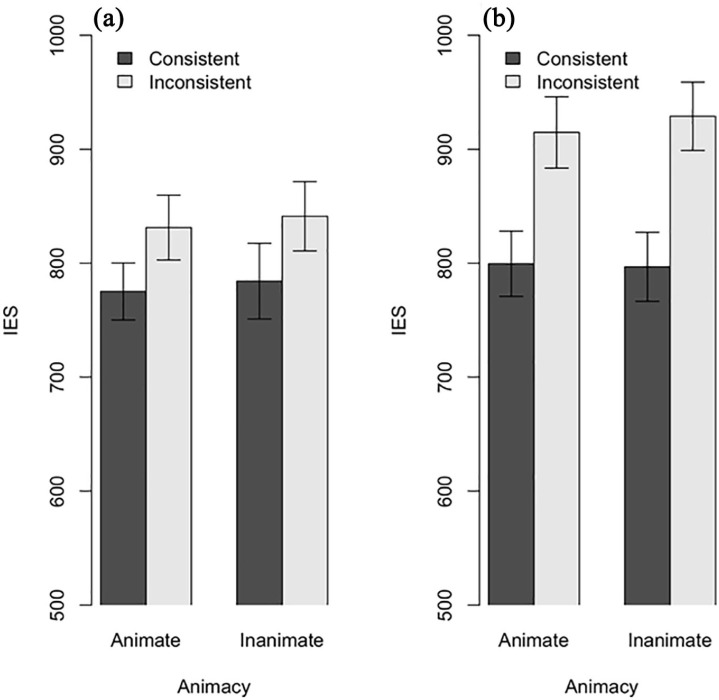
Experiment 2 Results for IES for (a) Self-perspective trials and for (b) Other-perspective trials. Error bars indicate standard error.

We conducted a three-way ANOVA, with Animacy, Perspective and Consistency as factors (see Figure 3). The results revealed a main effect of Consistency, *F*(1, 76) = 109.35, *p* < .001, $\eta_{p}^{2}=.056$ , with performance on consistent trials (*M* = 799.9, *SD* = 182.5) being superior to performance on inconsistent trials (*M* = 879, *SD* = 190.8). There was also a main effect of Perspective, *F*(1, 76) = 33.65, *p* < .001, $\eta_{p}^{2}=.019$ , with performance on self trials (*M* = 807.9, *SD* = 184.4) being superior to performance on other trials (*M* = 860, *SD* = 196.1). There was no main effect of Animacy, *F*(1, 76) = 0.04, *p* = .85, $\eta_{p}^{2}<.01$ , with performance not differing significantly between inanimate trials (*M* = 837.8, *SD* = 200.1) and animate trials (*M* = 830.2, *SD* = 183.7). We observed no significant interaction between Animacy and Perspective, *F*(1, 76) = 0.04, *p* = .83, $\eta_{p}^{2}<.001$ , and no significant interaction between Animacy and Consistency, *F*(1, 76) = 0.26, *p* = .61, $\eta_{p}^{2}<.001$ , whereas we did observe a significant interaction between Perspective and Consistency, *F*(1, 76) = 31.42, *p* < .001, $\eta_{p}^{2}=.01$ .

We also conducted a Bayesian analysis (using default priors and function stan_glmer() from the rstanarm package; see Goodrich et al., 2020) to quantify the support for the null effect of the interaction between Animacy and Consistency. Here, we found decisive evidence in support of the model in which there was no interaction, BF_01_ = 90.9. This means that there is roughly 91 times more evidence for the *absence* of an interaction effect than there is for the *presence* of an interaction effect.

As in Experiment 1, we observed a main effect of consistency. In Experiment 2, however, this effect was significant both when the task was to report what was in front of the novel entity (egocentric interference) and when the task was to report the content of one’s own perspective (altercentric interference).

The absence of any interaction between Animacy and Consistency in Experiment 2 is consistent with the hypothesis that the interference effect observed in Experiment 1 was due to the novel entity’s low-level directionality cues rather than its animacy. However, one possible confound of Experiment 2’s design is that participants in both conditions underwent the same familiarisation phase, which introduced both the animate and inanimate entities. Perhaps the highly visual similarity of the alien creature and mineral object created an “animacy carry-over” effect, leading them to represent the inanimate entity as having a perspective.

## Experiment 3

To minimise any “animacy carry-over” effect from the familiarisation phase to the test phase, Experiment 3 implemented a third version of the novel entity dot-perspective task. In this version, participants never learned that the inanimate entity was visually similar to any animate agent. That is, the familiarisation sequence only introduced the novel entity as a mineral formation. Participants did not learn about, or interact with, the animate alien that had been depicted in Experiments 1 and 2.

We predicted that if the null effect for Animacy observed in Experiment 2 was due to “animacy carry-over” from the alien to the mineral, we should not find a main effect of Consistency in Experiment 3. Such a finding would provide evidence that participants viewing the alien agent in Experiments 1 and 2 may yet have experienced interference that arose from taking its visual perspective. However, if there was no “animacy carry-over” effect in Experiment 2, then we should again observe an effect of Consistency in Experiment 3. This finding would provide evidence that participants experience interference on the dot-probe task arising from purely low-level directional cues. The hypotheses, sample sizes, methods, and initial analyses were all pre-registered before data collection. The pre-registration and the script used for this experiment, as well as all the raw data, can be accessed at: https://osf.io/2s6yc

### Participants

To facilitate comparison with the results of Experiments 1 and 2, we aimed for a sample size of 37 participants. We collected data from 63 participants, 24 of whom were excluded because their overall accuracy was below the threshold of 85%. Thus, the final sample was made up of 39 participants (20 males, 19 females, 0 undisclosed, *M_age_* = 20.77 years, *SD_age_* = 3.72 years). For recruitment, we used the participant database at the University of Warwick, where the experiment was conducted. All participants were naïve to the purpose of the study, reported normal or corrected-to-normal vision, and signed informed consent prior to the experiment. The experiment was conducted in accordance with the Declaration of Helsinki and was approved by Humanities and Social Sciences Research ethics committee. Each participant received gift vouchers totalling equivalent to €6 for participating.

### Apparatus and stimuli

The apparatus and stimuli were the same as in Experiment 2.

### Procedure

The procedure was identical to the procedure for the Inanimate condition in Experiment 2, except that participants underwent a different familiarisation phase that did not feature any entities described as animate, nor any entities with which participants directly interacted. Instead, participants clicked through a series of slides describing in some detail the physical properties of the unfamiliar entity, which was introduced as a mineral. This sequence used the same visual images as the full familiarisation sequence from Experiments 1 and 2 (with the exception of one slide in which we deleted a “thought bubble,” which would have otherwise functioned as a symbolic cue to animacy). Thus, the familiarisation in Experiment 3 was the same length as, and closely visually matched to, the familiarisation from Experiments 1 and 2.

### Results and discussion

As in Experiments 1 and 2, the mean HR in our sample was above 90% in all three conditions, indicating that it was appropriate to use IES for the analysis. In calculating mean RTs, response omissions due to the timeout procedure (1.64% of the data) and erroneous responses (4.62% of the data) were eliminated from the data set, as were trials where the correct response was “no” (50%). We also removed trials with responses that were more than 2.5 *SD*s greater or less than the mean for each participant for each condition (2.37% of the data).

**Figure 4.**
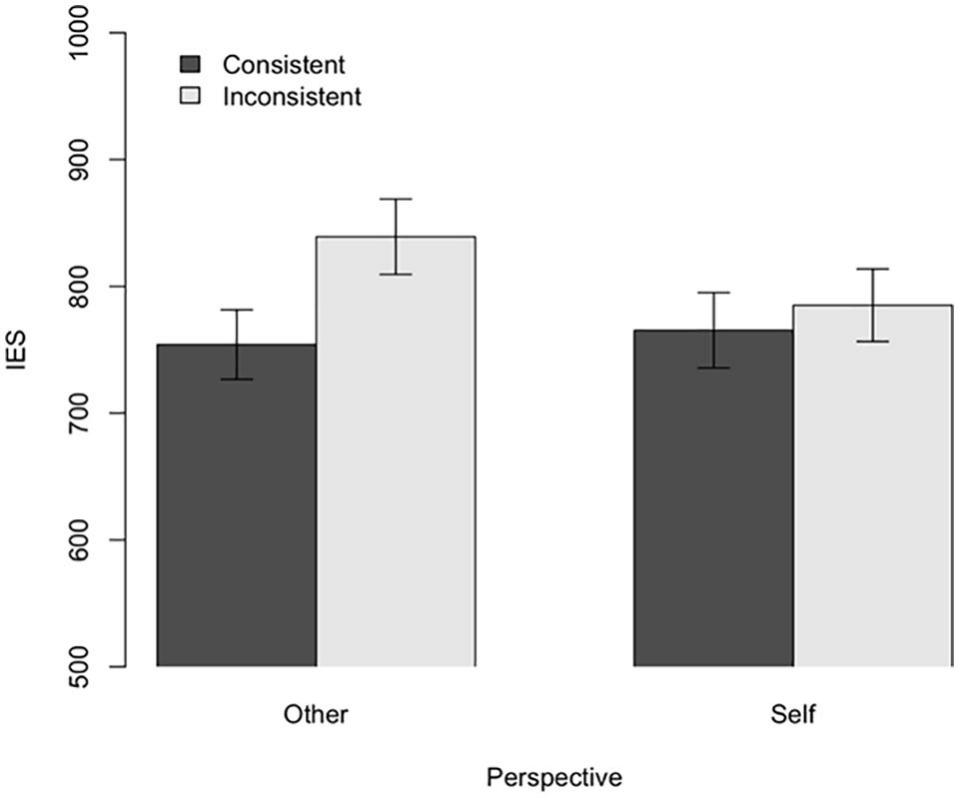
Experiment 3: Results for IES. Error bars indicate standard error.

We performed a two-way ANOVA for IES (see Figure 4). The results revealed a significant main effect of Consistency, with performance being better in the Consistent condition (*M* = 741.40, *SD* = 126.76) than in the Inconsistent condition (*M* = 819.29, *SD* = 144.10), *F*(1, 38) = 41.81, *p* < .001, $\eta_{p}^{2}=.08$ . There was also a main effect of Perspective—that is, performance was better in the Self condition (*M* = 755.36, *SD* = 120.61) than in the Other condition (*M* = 805.32, *SD* = 155.22), *F*(1, 38) = 14.09, *p* < .001, $\eta_{p}^{2}=.03$ . We also observed an interaction between perspective and consistency, *F*(1, 38) = 16.82, *p* *<* .001, $\eta_{p}^{2}=.02$ .

Planned contrast analyses revealed that the difference in performance between Consistent and Inconsistent trials was highly significant on Other trials, that is, when the task was to report what was in front of the novel entity, *t* (32) = 7.0, *p* < .001, *d* = 0.74; this provides evidence of egocentric interference. The difference in performance between consistent and inconsistent trials was also significant when the task was to report the content of their own perspective, *t*(38) = 3.41, *p* < .001, *d* = 0.37; this indicates evidence of altercentric interference.

In Experiment 3, we removed all discussion of animacy from the familiarisation phase to minimise the risk of any possible animacy carry-over effect. Nevertheless, we again observed a main effect of consistency (both altercentric and egocentric interference). This provides further support for the hypothesis that the effects observed in Experiments 1 and 2 were due to low-level directional cues instantiated by the central object.

## Experiment 4

Experiment 4 was designed to probe the possibility that the Consistency effect observed in the first three experiments may have been due to a design artefact. One notable feature of the dot-perspective task is that participants must constantly alternate between attending to and ignoring the directional orientation of the central figure, because they are instructed either to take its perspective or attend to its front. This raises the possibility that the interference effects on inconsistent Self trials were caused by difficulties with inhibiting a prepotent response to the direction of the stimulus, which would have been highly salient during Other trials (Conway et al., 2017, p. 56; Samson et al., 2010, p. 1259; Santiesteban et al., 2014, p. 934; Schurz et al., 2015, p. 387). This “task-switching hypothesis” amounts to a third possible explanation of the altercentric interference effect that we detected in earlier experiments: Unlike the perspective-taking hypothesis, it is not specifically triggered by the perceived perspectival characteristics of the central stimulus; unlike the standard submentalising account, the effect is not simply due to the fact that the stimulus has directional features to which participants display an automatic orienting response (Santiesteban et al., 2014, 2017) Rather, the task-switching hypothesis suggests that it is the challenge of alternating between attending to and ignoring the directionality of the central figure that drives altercentric interference. To test this possibility, this study employed a “Self-Only” design in which participants only ever completed trials in which they had to make judgements about their own perspective and were never asked to make judgements about the perspective/directionality of the novel entity (see Samson et al., 2010 and Santiesteban et al. 2014). This design eliminates any possibility of task-switching.

Another implication of the task-switching hypothesis is that it may have interfered with our animacy manipulation in Experiment 2. If task-switching is able to generate an altercentric interference effect on its own, this leaves open the possibility that an effect of animacy on altercentric interference might manifest in a task design that controls for this confound. That is, altercentric interference due to task-switching in our earlier inanimate conditions might have prevented us from detecting any effects of our animacy manipulation. Therefore, Experiment 4 also employed the same familiarisation and between-subjects animacy manipulation as was used in Experiment 2. This, combined with the aforementioned Self-only design, will enable us to test our initial study question about the respective roles of animacy and directionality in the dot-perspective task while at the same time controlling for the possibility of a task-switching confound. If the perspective-taking hypothesis is correct, we should expect to observe an interaction between Consistency and Animacy, with weaker performance in Inconsistent trials in the Animate condition only. If the submentalising hypothesis is correct, we should observe a main effect of Consistency, but no effect of Animacy. If, however, previously observed effects of Consistency were merely due to task-switching between Self and Other trials, then all Consistency effects should disappear in the current Self-only design.

One significant difference between the self-only and self-other designs is that it renders the animacy manipulation much more subtle. During Other trials in our previous experiments, participants were shown a screen that read either “Dax” or “Mineral,” which served as a repeated reminder of our Animacy manipulation. In the current Self-only design, however, this repeated reminder was absent, which opened up the possibility that participants might forget whether they are looking at an alien or a mineral (which are distinguished only by their colour). To guard against this possibility, this study employed an additional post-study manipulation check to ensure that participants in each condition actually represented the novel entity as either animate or inanimate.

The hypotheses, sample sizes, methods, and initial analyses were all pre-registered before data collection. The pre-registration and the script used for this experiment, as well as all the raw data, can be accessed at: https://osf.io/nwbrz.

### Participants

Using G*Power 3.1 (Faul et al., 2009), we determined that a sample size of 54 (27 per condition) would provide 95% statistical power for detecting an effect of *d* = 0.8 in the planned comparisons. For better comparison with Experiment 2, we decided to aim for a total of 72 participants. We collected data from 72 participants, 3 of whom were excluded because their overall accuracy was below the threshold of 85% and 13 of whom were excluded because they failed the manipulation check at the end. The COVID-19 pandemic made it unfeasible to replace these participants. Thus, the final sample was made up of 56 participants, 31 in the Animate condition and 25 in the Inanimate condition (33 males, 23 females, 0 undisclosed, *M_age_* = 26.5 years, *SD_age_* = 4.1 years).^2^ For recruitment, we used the participant database at the Central European University, where the experiment was conducted. All participants were naïve to the purpose of the study, reported normal or corrected-to-normal vision, and signed informed consent prior to the experiment. The experiment was conducted in accordance with the Declaration of Helsinki and was approved by the United Ethical Review Board for Research in Psychology (EPKEB). Each participant received gift vouchers totaling equivalent to €6 for participating.

### Apparatus and stimuli

The apparatus and stimuli were the same as in Experiments 1 and 2.

### Procedure

Participants viewed the same familiarisation as in Experiments 1 and 2. As in Experiment 2, one group of participants was informed that the entity in the virtual room was a mineral (Inanimate condition), and a separate group was informed that it was an animate agent (Animate condition). Within each group, half of the participants viewed a blue version of the novel entity, whereas the other half of the participants viewed a green version. Unlike in Experiment 2, participants in both conditions only completed Self trials in which they were asked to judge how many dots they could see from their own perspective, and never had to make any judgements about the “perspective” of the entity. After they completed the test trials, participants were redirected to an online post-study questionnaire containing a manipulation check asking participants to report whether the entity that they saw in the middle of the screen was an alien or a mineral; they could also select a third option indicating whether they did not remember. Participants were also asked to complete two open-ended response questions asking them to describe what they saw on the screen.

### Results and discussion

As in Experiments 1 and 2, the mean HR in our sample was above 90% in all three conditions, indicating that it was appropriate to use IES for the analysis. In calculating mean RTs, response omissions due to the timeout procedure and erroneous responses (3.09% of the data) were eliminated from the data set, as were trials where the correct response was “no” (50%). We also removed trials with responses that were more than 2.5 *SD*s greater or less than the mean for each participant for each condition (0.38% of the data).

**Figure 5.**
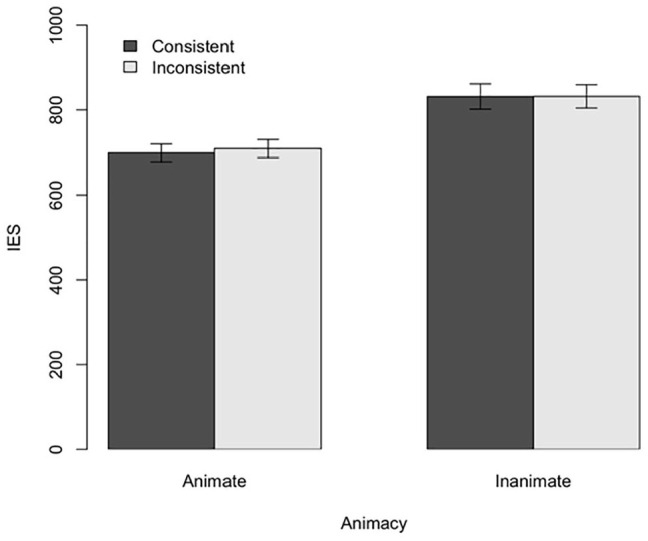
Experiment 4: Results for IES. Error bars indicate standard error.

We performed a two-way ANOVA for IES (see Figure 5). The results revealed no significant main effect of Consistency, *F*(1, 54) = 0.34, *p* = .56, $\eta_{p}^{2}<.01$ . There was a main effect of Animacy—that is, performance was better in the Animate condition than in the Inanimate condition, *F*(1, 54) = 14.3, *p* < .001, $\eta_{p}^{2}=.2$ . We did not observe a significant interaction between Animacy and Consistency, *F*(1, 54) = 0.32, *p* *=* .58, $\eta_{p}^{2}<.01$ .^3^

We also conducted a Bayesian analysis (again using default priors and function stan_glmer() from the rstanarm package) to quantify the support for the null effects of Consistency and the interaction between Animacy and Consistency. Here, we found decisive evidence in support of the model in which there was no main effect of consistency, BF_01_ = 66.7, and no interaction, BF_01_ = 66.7. This means that there is roughly 67 times more evidence for the *absence* of a main effect of consistency, as well as the interaction effect, than there is for the *presence* of an interaction effect.

Collectively, these results suggest that previous effects of Consistency were due to task-switching, rather than submentalising or perspective-taking. Contrary to the submentalising hypothesis, which explained previous results in terms of the directionality of the stimulus, we observed no effects of Consistency in this study. Contrary to the perspective-taking hypothesis, we did not observe any interactions between Animacy and Consistency. Surprisingly, we did observe a main effect of Animacy, albeit in the opposite direction as the one predicted by the perspective-taking hypothesis. Far from selectively interfering with performance, participants in the Animate condition actually exhibited superior performance. This may have been because construing the stimulus as animate made participants pay more attention to the task (Altman et al., 2016; New et al., 2007), but did not cause any sort of perspective-based interference.

## General discussion

We investigated whether prior findings of altercentric interference in a dot-perspective task are more likely to have arisen from mentalistic or submentalising cognitive processes. Unlike previous versions of the dot-perspective task that have relied upon highly familiar, intentionally laden inanimate controls to test these two competing accounts (e.g., Santiesteban et al., 2014), the central figure used in our dot-perspective task was a completely novel entity lacking agentic features. This enabled us to effectively manipulate whether participants viewed the central figure as an animate, perspectival agent or as an inanimate object with minimal directionality. In this way, we were able to more precisely test whether the altercentric interference effects traditionally found in the dot-perspective task are due to representations of perspectival agency (as predicted by the mentalising hypothesis) or due to low-level directional properties of the stimulus (as predicted by the submentalising hypothesis).

In Experiments 1–3, we observed altercentric interference in this new version of the dot-perspective task. Experiment 1 provided an initial validation of our novel stimuli. In Experiment 2, we manipulated whether participants were informed either that the central entity was animate (an alien) or that it was inanimate (a mineral formation). In this context, the mentalising hypothesis predicts that only participants instructed to view the entity as an agent should display the altercentric interference effect, because this is the only condition in which the central stimulus is assumed to have a visual perspective. In contrast, the submentalising hypothesis predicts that participants instructed to view the entity as an inanimate object should also display such an effect. We repeatedly found main effects of Consistency, but no main effects of Animacy or interactions between Consistency and Animacy, which was most consistent with the submentalising hypothesis. This interpretation was further supported by the results of Experiment 3, which also found an effect of Consistency when participants viewed the central entity as an inanimate object.

Our final experiment, however, led us to view these earlier experiments in a different light. Experiment 4 was designed to probe the possibility that the Consistency effect observed in the first three experiments might have been due to the demands of task-switching across Self and Other trials. During Other trials, the directionality of the central figure is task-relevant and therefore highly salient. During Self trials, these directional features are not task-relevant. However, due to the need to constantly alternate between Self and Other trials, the directional properties of the central figure very likely retain their salience for the participants throughout the experiment. Altercentric interference effects on Self trials might therefore stem from difficulties with inhibiting a prepotent response to directional features made salient by the task demands of Other trials. In other words, altercentric interference effects may result from task-switching, rather than any “automatic” or “spontaneous” tendency to engage in perspective-taking or directional orienting. The results of Experiment 4, in which we only included “Self” trials and observed no effect of Consistency, support this interpretation.

Notably, other implementations of the dot-perspective paradigm have discovered altercentric interference effects in Self-only designs. Samson et al. (2010) employed a Self-only design using both a human avatar and a rectangle as an inanimate control and found a significant interaction between Consistency and their Animacy variable, which they interpreted as supporting the perspective-taking hypothesis. As Santiesteban et al. (2014) pointed out, however, the rectangle was not a well-matched inanimate control because it lacked strong directional characteristics. They implemented a Self-only design that compared a human avatar with an arrow and found a Consistency effect but no evidence of an interaction between Consistency and Animacy, which they interpreted as supporting the submentalising hypothesis. In their Self-only version of Furlanetto and colleagues’ “goggles test” dot-perspective task, which used human avatars, Conway et al. (2017) were also able to obtain an effect of Consistency; again, this was interpreted as supporting the submentalising hypothesis. All considered, a pattern emerges: Human avatars and arrows each appear to reliably generate altercentric interference effects even in Self-only designs, while the novel entity avatar used in the current experiments only generated these effects in a Self-Other design. On the one hand, this tells us that task-switching is sufficient to generate altercentric interference effects even with very novel stimuli with minimal directional characteristics, regardless of whether these stimuli are construed as perspectival agents; on the other hand, more familiar stimuli like arrows and human avatars seem to be sufficient to generate these effects as well, independently of any task-switching effects.

These conclusions have challenging implications for the mentalising/submentalising debate. As noted in the introduction, earlier attempts to tease apart these hypotheses using arrows as inanimate controls were unsatisfactory because arrows still confound directionality with animacy because of the arrows’ derived intentionality (Searle, 1983). In the current studies, we eliminated this confound by using a novel entity as an avatar and manipulated the perceived animacy of the stimulus while leaving its directionality fixed. When deployed in a Self-Other design, this stimulus generated a pattern of results that seemed to support the submentalising hypothesis. But once we eliminated the task-switching confound and the directionality of the novel entity was no longer task-relevant, this pattern of results disappeared. This suggests that, contrary to the submentalising hypothesis, *mere* directionality—that is, having a “front”—is not by itself enough to explain the full pattern of results that we see in various versions of the dot-perspective task. In addition, the fact that our animacy manipulation also failed to induce any altercentric interference effects independent of task-switching suggests that mere beliefs about the animacy of a stimulus are *also* not enough to produce this effect, contrary to the mentalising hypothesis. In short, the present results support *neither* the mentalising hypothesis *nor* the submentalising hypothesis, at least as it has been typically understood in this task.

Our results could, however, be interpreted as supporting a more generic version of the submentalising hypothesis that simply denied any particular role for domain-specific perspective-taking in the dot-perspective task (e.g., Heyes, 2014). As the proposed task-switching account invokes only domain-general processes, it could perhaps be regarded as a form of submentalising in this looser sense. However, it remains incompatible with the standard submentalising explanation of the dot-perspective task outlined in Santiesteban et al. (2014) and Santiesteban et al. (2014), which focuses on automatic directional orienting. One implication of this interpretation is that there may be many different forms of submentalising. Researchers aiming to test whether a given task involves mentalising or submentalising should therefore take care to specify which submentalising process they think might be at work.

Speculatively, the conditions under which altercentric interference effects do emerge all seem to share a common feature: Something about the directionality of the stimulus made it *relevant* or *salient* to the participant, either because it was a person, a highly familiar symbol, or because they had recently attended to it in the context of another task. This observation recalls a distinction between perspective-selection and perspective-calculation introduced by Qureshi et al. (2010) and discussed in Westra (2017). Based on evidence from a version of the dot-perspective task that used a dual-task interference manipulation, Qureshi et al. proposed that the “perspective-taking” process could be broken down into a perspective-selection process that recruits top-down attentional control to select a particular agent for a subsequent, automatic perspective-calculation process (i.e., determining what falls within that agent’s line of sight). Building on this idea, Westra (2017) argued that the perspective-selection component of this process is likely to be supported by goal-sensitive, unencapsulated orienting systems—specifically, the ventral attention network, which detects salient or task-relevant stimuli (Corbetta et al., 2008). If this breakdown of the process underlying performance in the dot-perspective task is correct, it implies that the systems controlling the inputs to the directionality-sensitive “perspective”-calculation process are entirely domain-general and will respond to whatever stimuli happen to be behaviorally relevant. Some highly familiar stimuli, like human figures and arrows, might be treated as relevant by default, whereas other less familiar stimuli (like our novel entity) might only engage these salience systems in the contexts of certain behavioural tasks.

This picture lends itself to a hybrid model of visual perspective-taking that makes room for both domain-general and domain-specific processes (Michael & D’Ausilio, 2015). According to this hybrid approach, the discovery that domain-general processes are involved in visual perspective-taking need not preclude the possibility that domain-specific social representations are also involved. Instead, it might be that domain-specific perspective-taking processes are implemented in part by domain-general spatial cueing mechanisms that are also triggered by non-social stimuli. In other words, although visual perspective-taking and spatial cueing might involve overlapping sets of processes and may manifest similar behavioural profiles, they might also involve non-overlapping sets of processes, which might be distinguished through precisely targeted experimental designs (e.g., Friesen & Kingstone, 1998; Marotta et al., 2012). Identifying which domain-general processes are involved in social perception could thus play a positive, structuring role in the way that domain-specific perspective-taking processes are modelled.

The current set of studies was limited in a few ways. One limitation of Experiments 1–3 was that they did not employ any manipulation checks to ensure that participants really construed the novel entity as animate or inanimate. Given this limitation, it could be argued that we cannot be certain whether our animacy manipulation was truly successful: Participants might have confused the mineral with the alien or forgotten which was which. However, the nature of the Other trials in Experiments 1–3 ensured that participants were repeatedly reminded that the novel entity was either an alien or a mineral, making this possibility unlikely. Experiment 4 lacked these repeated reminders, and so we did include a manipulation check in that study, which led to the exclusion of a number of participants who could not be replaced due to public health restrictions on data collection. Even with these exclusions, we were able to detect a significant effect of Animacy.

A further limitation of the current set of studies concerns the high number of excluded participants in Experiments 2 and 3 because they did not meet our minimum performance threshold. This was likely due to the fact that participants in these two studies were tested in groups rather than individually. The presence of other people in the testing environment may have caused some participants to become distracted, which may have affected their performance and resulted in their exclusion. This suggests that a future best practice for this paradigm may be to avoid testing participants in groups.

Overall, we found compelling evidence that a completely novel entity can trigger an altercentric interference effect in a dot-perspective task and no evidence that beliefs about the animacy of this entity modulate this effect, contrary to the mentalising hypothesis. However, we also found evidence that this effect was not due solely to the directional properties of the stimulus, which is inconsistent with standard versions of the submentalising hypothesis. We interpret this set of results as showing that the mechanisms underlying the altercentric interference effect are influenced by salience systems that are sensitive to the perceived relevance of the directional stimulus, either in the context of the task itself (as with our novel entity) or because of their familiarity as directional cues (as we see in previous studies employing humans and arrows). This suggests that whatever causes the altercentric interference effect, it is likely to be gated by domain-general attentional systems, and thus sensitive to a wide range of stimuli, beyond avatars and arrows.

## Notes

1. The relatively high exclusion rate for this Experiment (and the next one) may have been due to the fact that for this study, participants were tested in groups, whereas in Experiment 1 they were tested one at a time. See the “General Discussion” section for more on this potential limitation of this study.

2. Supplemental analyses conducted without these exclusions (*N* = 69) did not affect any of the primary results of this study.

3. Although we also collected responses to additional two open-ended post-study survey questions, the analysis of these data was rendered moot by the fact that we did not discover any altercentric interference effects. As such, we elected not to analyse these responses. We do include them on the OSF project page: https://osf.io/9ghvs/files/.
